# Switching pattern and dose adjustment of antidepressants before and during pregnancy

**DOI:** 10.1007/s00737-023-01355-8

**Published:** 2023-08-05

**Authors:** Robiyanto Robiyanto, Marjolein Roos, Jens H J Bos, Eelko Hak, Eugène P van Puijenbroek, Catharina C M Schuiling-Veninga

**Affiliations:** 1 Unit of PharmacoTherapy, Epidemiology, & Economics, Groningen Research Institute of Pharmacy, University of Groningen, Groningen, The Netherlands; 2 Program Studi Farmasi, Fakultas Kedokteran, Universitas Tanjungpura, Pontianak, Indonesia; 3 Netherlands Pharmacovigilance Centre Lareb, ‘s-Hertogenbosch, The Netherlands

**Keywords:** Antidepressants, Dose adjustment, Pregnancy, Switching pattern

## Abstract

The purpose of the study is to examine the switching pattern and dose adjustment of antidepressants (ADs) prescribed to women from six months before to six months during pregnancy in the Netherlands. The recorded dispenses or refills were collected from the University of Groningen IADB.nl pregnancy subset for all singleton pregnancies in which the mother received ≥ 1 prescription of an AD dispensed before pregnancy and was present in the database at least six months after conception. The rates of continuation, discontinuation, and switching between 2001 and 2020 were assessed for the ADs studied. The mean number of Defined Daily Doses (DDDs) of the most frequently continued ADs used was calculated both before and during pregnancy, and a paired *t*-test was used to test for significant changes. The continuation rates for AD users, especially for SSRI and SNRI continued users, increased over time from 27% and 19% (2001–2005) to 65% and 65% (2016–2020). The switching rate between ADs remained consistently low from the start of the study (2001–2005) at 2.0% to the end of the study (2016–2020) at 2.3%. Most women who switched between antidepressants during pregnancy received a different SSRI monotherapy (85%), followed by an SNRI (6%), a TCA (4%), and an “other AD” (4%). In most cases observed, the dose adjustment for the mean DDDs during pregnancy compared to the mean DDDs before pregnancy only changed little (less than 10%). Continued use of SSRIs among singleton pregnancies doubled over the study period. The low rate of AD switching and little changes in the DDD adjustment for most AD continuers indicate that pregnant women prefer to continue their prepregnancy medication rather than switch it. Most observed findings cohere with the Dutch national guidelines for antidepressant use during pregnancy.

**Supplementary Information:**

The online version contains supplementary material available at 10.1007/s00737-023-01355-8.

## Introduction

Depression during pregnancy is estimated to affect approximately 16.4% of women worldwide and the efficacy of antidepressants (ADs) has been proven, especially in combination with psychotherapy (Arroll et al. 2009; Okagbue et al. 2019). The global prevalence of AD use during pregnancy was estimated at 3% for selective serotonin reuptake inhibitors (SSRIs), followed by serotonin and norepinephrine reuptake inhibitors (SNRIs) at 0.73% and tricyclic antidepressants (TCAs) at 0.38% (Molenaar et al. 2020a). The indications of use for ADs during pregnancy are notably for depressive or anxiety disorders (Zorginstituut Nederland 2022a, 2022b). Untreated perinatal depression or anxiety disorders during pregnancy appear to be unfavorable and inevitable, including the risk of relapse, preterm birth, low neonatal weight, and postnatal complications (ACOG 2008).

In the Netherlands, the national guideline on the use of SSRI and non-SSRI antidepressants during pregnancy was initiated by the Dutch Association for Obstetrics and Gynecology (“Nederlandse Vereniging voor Obstetrie en Gynaecologie” (NVOG)) together with the Dutch Associations for Pediatrics (“Nederlandse Vereniging voor Kindergeneeskunde” (NVK)) and the Dutch Association for Psychiatry (“Nederlandse Vereniging voor Psychiatrie” (NVvP)) (NVOG 2012, 2021). Before the NVOG guideline was published in 2012, the first advice on ADs during pregnancy had been mentioned in 2005 by the Teratology Information Service of the National Pharmacovigilance Centre (Lareb) in the “Commentaren Medicatiebewaking” used by physicians and pharmacists as a practical reference book when prescribing and dispensing medicines (Stichting Health Base 2020). Both existing recommendations from NVOG and Lareb explain that there is no solid evidence for discontinuing ADs during pregnancy based on the risk they pose when the mother is stable and well-adjusted to the medication they are taking compared to the possible risk of relapse (NVOG 2012; Bijwerkingen Centrum Lareb 2021a). In any case, stopping or switching medication abruptly during pregnancy is not advised due to increased relapse/recurrence risk (Bijwerkingen Centrum Lareb 2021a, 2022; NVOG 2021). Detailed information for which AD medication should be switched and which AD requires a dose adjustment during pregnancy is not explicitly stated in both recommendations.

In 2018, a study in France reported that switching between ADs occurred in 9.1% of all exposed pregnancies, and sertraline was mentioned as the most switched-to drug, followed by es-/citalopram (Bénard-Laribière et al. 2018). In the Netherlands, switching patterns and dose adjustments of ADs around pregnancy remain insufficiently explored. In this study, we aimed to elucidate trends in continuing use of ADs, switching patterns between ADs, and DDD adjustment of AD prescription before and during pregnancy. The findings were compared to the existing professional advice in the Netherlands to evaluate if there is an alignment between the observed trends and the Dutch national guidelines.

## Methods

### Setting, study population

A retrospective drug utilization study was performed using the pregnancy subsection of the University of Groningen IADB.nl longitudinal database for recorded dispenses (IADB.nl 2022). The general population in the IADB.nl was reported to represent the Dutch population overall (Visser et al. 2013; Sediq et al. 2018). From 1994 to 2021, this database had more than 2.7 million prescriptions dispensed from over 120 community pharmacies which covers more than 1.2 million people residing in the northeastern Netherlands. The prescription dispensed in the IADB.nl includes information, e.g., dispensing date, quantity and duration of drug prescribed, dose regimen, drug prescriber, and drug’s Anatomical Therapeutic Chemical (ATC) code. Dispensing data from hospital pharmacies or over-the-counter (OTC) medication are not recorded in the IADB.nl (IADB.nl 2022). The pregnancy subset in the IADB.nl uses a linkage/coupling approach in which a mother is linked to her child (both are anonymous patients) based on the address code and the mother’s age at delivery. This strategy was considered to be a valid method (Schirm et al. 2004).

The study population included all singleton pregnancies having ≥1 dispensing of AD (ATC code starting with N06A) (WHOCC 2022a) recorded within six months prior to pregnancy and were present in the database at least during six months after Theoretical Conception Date (TCD). The first pregnancy date or TCD was set at 39 weeks (13 weeks per trimester) before the child’s birth date. The study period covered all pregnancies from 1^st^ January 2001 to 31^st^ December 2020. A woman who had multiple pregnancies during the study period could be included more than once if in each pregnancy a woman had at least 1 prescription dispensed recorded in the six months before pregnancy. ADs with low number of users (≤2 pregnancies exposed) were excluded from the analysis.

### Continuation, discontinuation, and switching of antidepressants before and during pregnancy

Continued users were defined as pregnancies with ≥1 prescription of an AD dispensed before pregnancy and ≥2 prescriptions of the same AD dispensed during pregnancy. Pregnancies with ≥1 AD prescription dispensed before pregnancy, ≤1 prescription of the same AD dispensed during pregnancy, and no switch to another AD during pregnancy were considered discontinued users. Switched users were defined as pregnancies with ≥1 prescription dispensed before pregnancy, ≤1 prescription of the same AD dispensed during pregnancy, and ≥2 prescriptions of another AD dispensed during pregnancy. A woman with a combination of two ADs could be a continuer of one AD and a discontinuer of another.

The ratio between the number of continued users and the total users was calculated as the continuation rate per overall study period (2001–2020) and per five years (2001–2005, 2006–2010, 2011–2015, 2016–2020). Next, within the SSRI, SNRI TCA, and other AD classes, the percentage of users who continue, discontinue, and switch between individual ADs was evaluated. Finally, the patterns of switching between ADs before and during pregnancy were evaluated for each class and medication.

### DDD adjustment of antidepressants before and during pregnancy

To assess if there is an adjustment in the daily dose used of an AD before and during pregnancy, we included the top five ADs with the most continued users by comparing the mean number of DDDs used per day before and during pregnancy. The absolute difference between these two means was measured by subtracting the mean number of DDDs used before with the mean number of DDDs used during pregnancy for each AD. The DDD adjustment (%) was calculated as the proportion of the absolute difference of number of DDDs divided by the mean number of DDDs used before pregnancy. The positive result (in %) indicates an increase in the mean number of DDDs used of an AD prescription during pregnancy and vice versa.

To assess whether an adjustment in the number of DDDs of an AD differed for those who initially used a high or low dose before and during pregnancy, since there is no specific dose advice for pregnant women, we used the cut-off values based on the recommended dose which is 1 DDD. The mean and median doses used of all almost ADs studied were also close to 1 DDD per day, except for sertraline in which the mean and median doses were around 1.5 DDD per day. Thus, we used 1.5 DDD as the cut-off point for sertraline users.

By using 1 (or 1.5) DDD as the cut-off value, we distinguish if a woman taking an AD before pregnancy belonged to the low-dose group (if using <1 DDD per day) or the high-dose group (using ≥ 1 DDD per day). The higher mean and median doses for sertraline users might be influenced by the wide range of sertraline recommended doses for adults: 50 to max 200 mg/day, equivalent to 1–4 DDD (WHOCC 2022b; Zorginstituut Nederland 2022c).

### Statistical analysis

The significant difference in the mean age between the singleton pregnancy population and the AD users before pregnancy was analyzed using an independent *t*-test, while the significant difference in the mean DDDs of an AD prescribed before and during pregnancy was analyzed using a paired *t*-test. A two-sided significance threshold used was *P* <0.05. All statistical analyses used IBM SPSS V28.0 (Armonk, NY, USA).

## Results

**Fig. 1:**
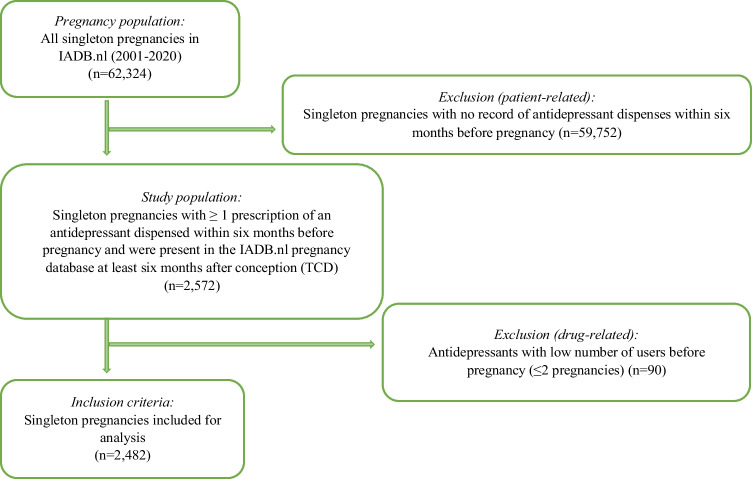
Cohort selection flowchart

In this study, 2482 singleton pregnancies with at least one dispensing of an AD before pregnancy were included. These pregnancies comprised 2179 women (mothers) with one or multiple pregnancies that met our cohort inclusion criteria. Among 2482 pregnancies, 129 were prescribed two ADs and 22 with three ADs, giving 2655 times an AD was prescribed as prepregnancy medication. ADs with low number of users (≤2 pregnancies) before pregnancy were excluded from the cohort selection (S1 Table). The cohort selection flowchart is shown in Fig. 1.

Table 1 shows that the difference between the mean SD age (years) of singleton pregnancies who received AD medication before pregnancy (2482 pregnancies) is statistically significant than that of all singleton pregnancies’ population (62,324) in the IADB.nl. From 2006 to 2020, the mean age of exposed pregnancies is always older than the mean age of the pregnancy population but the absolute age difference between the groups was smaller (more less 1 year). The exposure rate to AD drugs among singleton pregnancies studied showed an increased trend from 3.1% (2001–2005) to 4.8% (2016–2020). ADs with the most number of users (*n*) within six months before and six months during pregnancy were represented by paroxetine (573), citalopram (516), amitriptyline (326), sertraline (251), and venlafaxine (224).

**Table 1:** General characteristics of the antidepressant users in the IADB.nl pregnancy database

	*Total population of singleton pregnancies*		*Antidepressant users before pregnancy*				*Antidepressants with the most number of users before and during pregnancy*				
	*n*	*Mean age (years ± SD)*	*n*	*Mean age (years ± SD)*	*Exposure rate (%)*	*P value*	*Paroxetine n (%)*	*Citalopram n (%)*	*Amitriptyline n (%)*	*Sertraline n (%)*	*Venlafaxine n (%)*
*Total study period*	62,324	29.4 ± 4.7	2482	30.1 ± 4.8	4.0	<0.0001*	573 (100)	516 (100)	326 (100)	251 (100)	224 (100)
*2001*–*2005*	13,561	29.5 ± 4.7	420	29.9 ± 5.1	3.1	0.087	165 (28.8)	46 (8.9)	61 (18.7)	19 (7.6)	37 (16.5)
*2006*–*2010*	14,806	29.4 ± 4.7	553	30.0 ± 5.1	3.7	0.003*	178 (31.1)	82 (15.9)	61 (18.7)	27 (10.8)	68 (30.4)
*2011*–*2015*	19,247	29.2 ± 4.6	804	29.9 ± 4.8	4.2	<0.0001*	148 (25.8)	196 (38.0)	109 (33.4)	82 (32.7)	78 (34.8)
*2016*–*2020*	14,710	29.5 ± 4.5	705	30.7 ± 4.4	4.8	<0.0001*	82 (14.3)	192 (37.2)	95 (29.1)	123 (49.0)	41 (18.3)

### Continuation, discontinuation, and switching rates of antidepressants before and during pregnancy

**Fig. 2:**
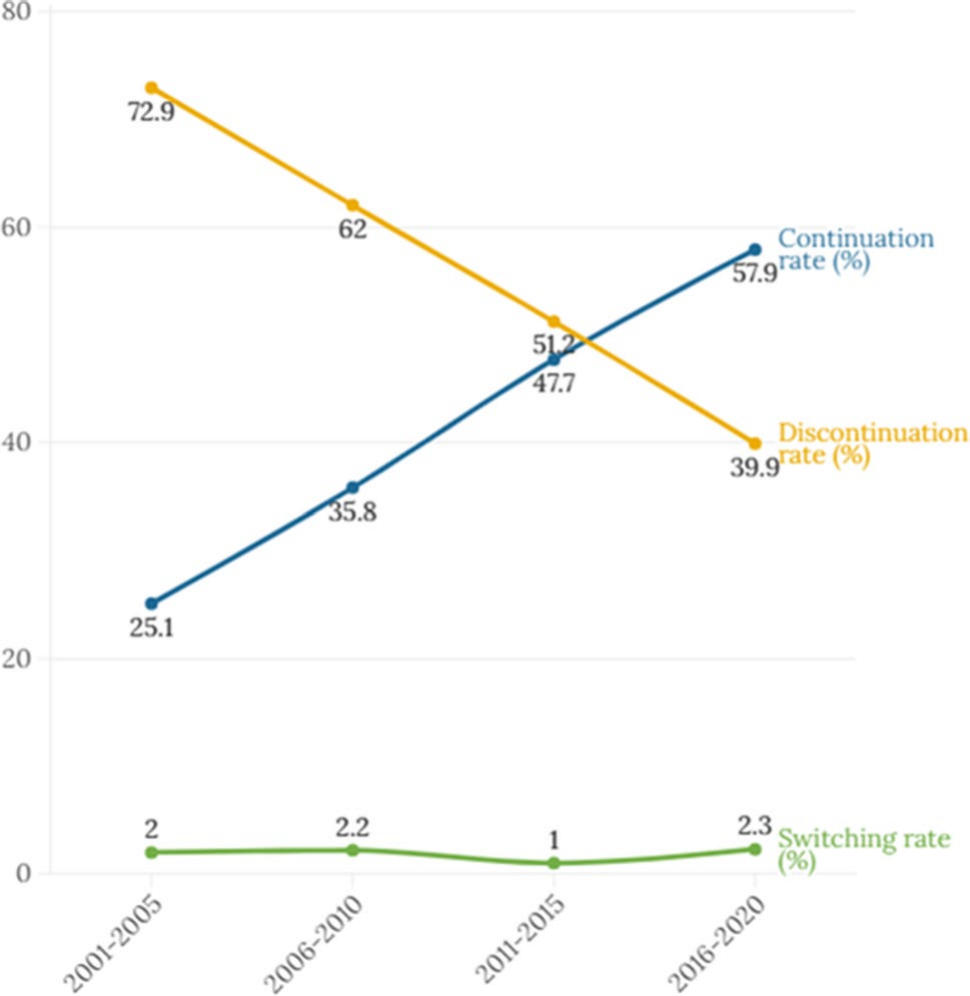
Continuation, discontinuation, and switching rates of antidepressants before and during pregnancy from 2001 to 2020

The change in the continuation, discontinuation, and switching rates of ADs per five years between 2001 and 2020 is illustrated in Fig. 2. The continuation rate for all ADs increased over time from 25.1% (2001–2005) to 57.9% (2016–2020), whereas the discontinuation rate decreased from 72.9% at the start to 39.9% at the end of the study period. The switching rate between antidepressants was consistently low over time with a little increase from 2.0% in 2001–2005 to 2.3% in 2016–2020 (S2 Table).

When stratifying the AD users per class, the proportion of women who continued ADs from SSRI and SNRI classes during pregnancy raised from 27% and 19% (2001–2005) to 65% and 65% (2015–2020). In contrast, the proportion of women who discontinued the use of TCAs and “other ADs” remained high (>60%) during the observed period (Table 2). Regarding the continued use of individual ADs, the continuation rates of sertraline (5 to 62%) and citalopram (26 to 71%) showed an increasing trend per five years of observation. Bupropion (84%), nortriptyline (80%), and amitriptyline (78%) had the highest proportion of discontinuers among all TCAs and “other ADs” being discontinued during pregnancy in the overall period. The switching rate for each class and individual ADs were based on the number of users where ADs being switched to during pregnancy. In Table 2, we can see that fluoxetine, sertraline, and citalopram had a higher proportion of switchers than other ADs.

**Table 2:** Proportion^$^ of continuers, discontinuers, and switchers of antidepressant medication^#^ over the total study period and per five years per individual drug before and during pregnancy

*Antidepressants*	*% Continuers (n)*					*% Discontinuers (n)*					*% Switchers (n)*				
	*2001–2005*	*2006–2010*	*2011–2015*	*2016–2020*	*Total period*	*2001–2005*	*2006–2010*	*2011–2015*	*2016–2020*	*Total period*	*2001–2005*	*2006–2010*	*2011–2015*	*2016–2020*	*Total period*
***SSRI***	27 (81)	41 (155)	53 (296)	65 (337)	50 (869)	71 (212)	56 (211)	45 (251)	33 (169)	48 (843)	2 (7)	3 (11)	2 (9)	3 (14)	2 (41)
*Citalopram*	26 (12)	30 (25)	51 (100)	71 (137)	53 (274)	74 (34)	67 (55)	48 (95)	26 (50)	45 (234)	–	2 (2)	1 (1)	3 (5)	2 (8)
*Paroxetine*	30 (50)	45 (80)	55 (81)	68 (56)	47 (267)	68 (112)	53 (94)	45 (66)	32 (26)	52 (298)	2 (3)	2 (4)	1 (1)	–	1 (8)
*Sertraline*	5 (1)	33 (9)	61 (50)	62 (76)	54 (136)	95 (18)	63 (17)	37 (30)	33 (41)	42 (106)	–	4 (1)	2 (2)	5 (6)	4 (9)
*Fluoxetine*	22 (11)	48 (26)	53 (35)	49 (18)	43 (90)	70 (35)	44 (24)	41 (27)	49 (18)	50 (104)	8 (4)	7 (4)	6 (4)	3 (1)	6 (13)
*Escitalopram*	–	35 (7)	43 (21)	56 (45)	49 (73)	–	65 (13)	57 (28)	42 (34)	50 (75)	–	–	–	2 (2)	1 (2)
*Fluvoxamine*	35 (7)	50 (8)	60(9)	100 (5)	52 (29)	65 (13)	50 (8)	33 (5)	–	46 (26)	–	–	7 (1)	–	2 (1)
***SNRI***	19 (7)	45 (34)	62 (57)	65 (33)	51 (131)	78 (29)	54 (41)	38 (35)	33 (17)	48 (122)	3 (1)	1 (1)	–	2 (1)	1 (3)
*Venlafaxine*	19 (7)	46 (31)	69 (54)	66 (27)	53 (119)	78 (29)	53 (36)	31 (24)	32 (13)	46 (102)	3 (1)	1 (1)	–	2 (1)	1 (3)
*Duloxetine*	–	38 (3)	21 (3)	60 (6)	38 (12)	–	63 (5)	79 (11)	40 (4)	63 (20)	–	–	–	–	–
***TCA***	24 (19)	17 (14)	32 (49)	36 (44)	29 (126)	74 (58)	82 (68)	68 (103)	64 (77)	71 (306)	1 (1)	1 (1)	–	–	1 (2)
*Amitriptyline*	15 (9)	10 (6)	21 (23)	35 (33)	22 (71)	85 (52)	90 (55)	79 (86)	65 (62)	78 (255)	–	–	–	–	–
*Clomipramine*	50 (6)	41 (7)	92 (22)	90 (9)	70 (44)	42 (5)	53 (9)	8 (2)	10 (1)	27 (17)	8 (1)	6 (1)	–	–	3 (2)
*Nortriptyline*	100 (2)	–	22 (4)	13 (2)	20 (8)	–	100 (4)	78 (14)	88 (14)	80 (32)	–	–	–	–	–
*Others*	66 (2)	100 (1)	–	–	60 (3)	33 (1)	–	100 (1)	–	40 (2)	–	–	–	–	–
***Other AD***	17 (6)	13 (6)	17 (11)	37 (23)	22 (46)	83 (30)	88 (42)	83 (54)	60 (38)	77 (164)	–	–	–	3 (2)	1 (2)
*Mirtazapine*	26 (6)	9 (3)	23 (9)	37 (13)	23 (31)	74 (17)	91 (31)	78 (31)	60 (21)	76 (100)	–	–	–	3 (1)	1 (1)
*Bupropion*	–	–	6 (1)	40 (8)	16 (9)	100 (11)	100 (9)	94 (16)	60 (12)	84 (48)	–	–	–	–	–
*Others*	–	60 (3)	13 (1)	25 (2)	26 (6)	100 (2)	40 (2)	88 (7)	63 (5)	70 (16)	–	–	–	13 (1)	4 (1)

^$^The continuers (%) + discontinuers (%) + switchers (%) add up to 100% for each class and each individual ADs

^#^AD drugs with a low number of users (<10 pregnancies) were not displayed individually but combined as “*others*”

*Number of switchers is based on the antidepressant being switched to during pregnancy

### Switching patterns between antidepressants before and during pregnancy

**Fig. 3:**
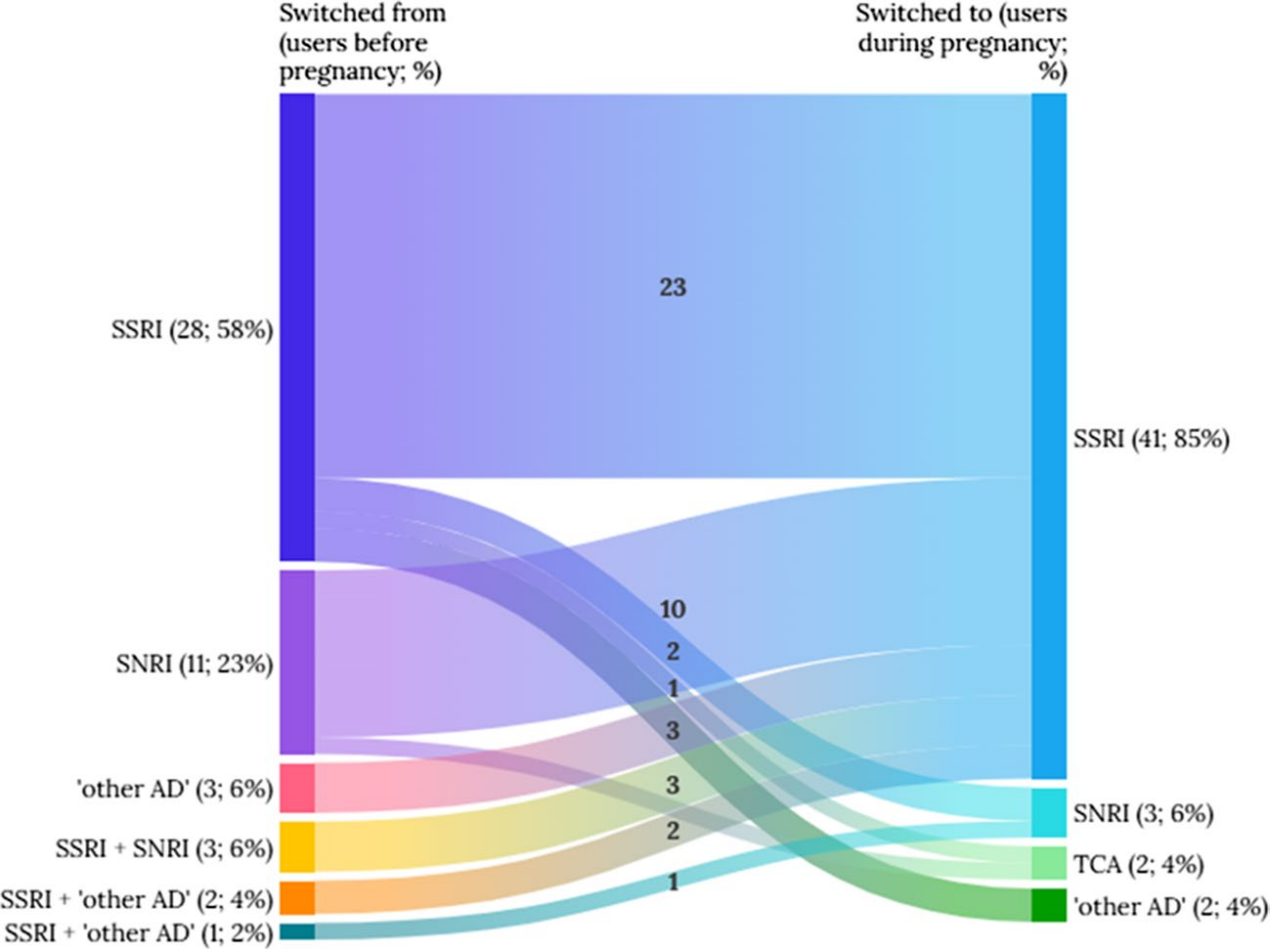
Switching patterns between antidepressants before and during pregnancy from 2001 to 2020 (*n*=48 switchers)

Among all switchers of antidepressants (*n*=48) observed, 42 women (87.5%) used AD monotherapy and the rest received AD combinations before pregnancy, after which all of them switched to another AD monotherapy during pregnancy (Table 3). The AD combinations always consisted of concomitant use of SSRI with either SNRI or “other AD.” 23 out of 28 women (82%) who already used an SSRI before pregnancy switched to a different SSRI during pregnancy (Fig. 3). Almost all who previously used an SNRI, an “other AD,” or AD combinations switched to an SSRI during pregnancy. The switched-to ADs were dominated by an SSRI monotherapy (85%), followed by an SNRI (6%), a TCA (4%), and an “other AD” (4%).

**Table 3:**
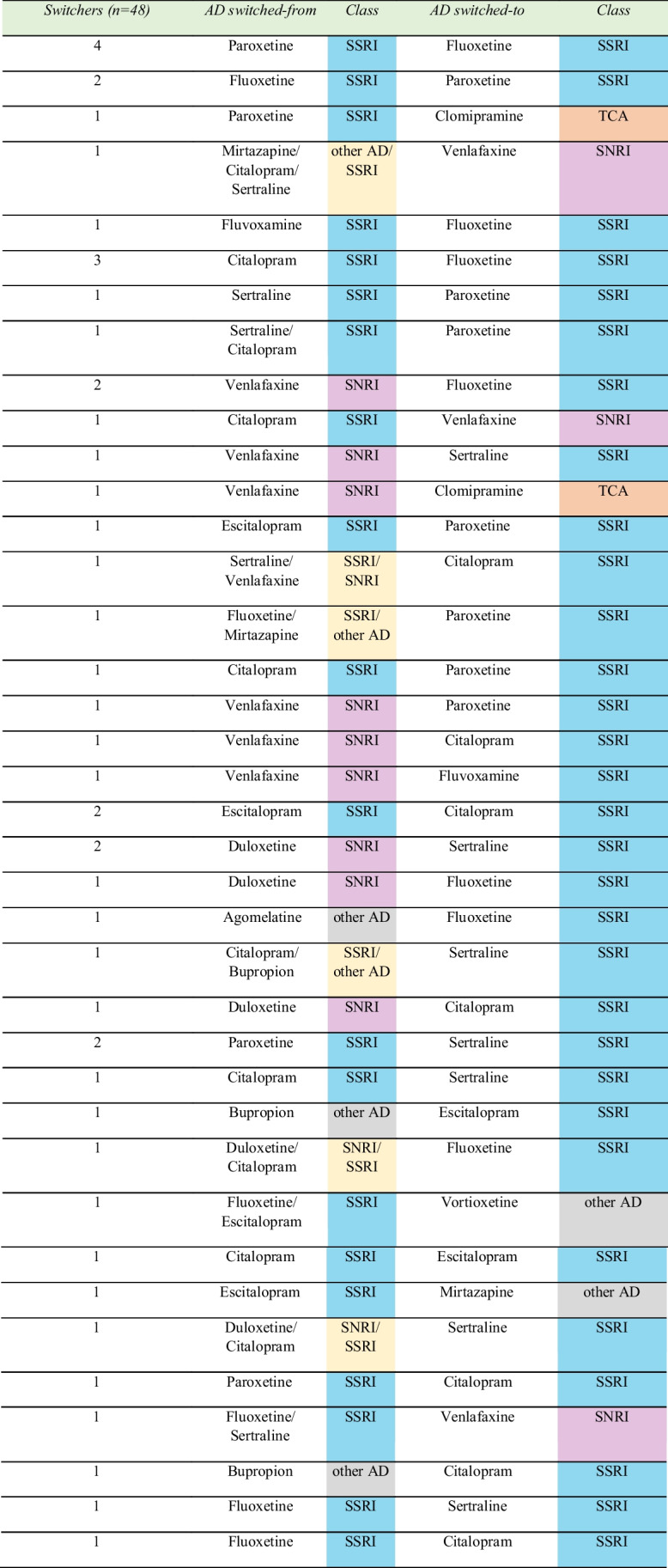
Switching of antidepressant drugs within continued users group (*n*=48) from 2001 to 2020

In respect to the switching patterns, citalopram (12 users), paroxetine (8 users), and venlafaxine (8 users) were the most switched-from ADs, whereas fluoxetine (13 users) and sertraline (9 users) were the most popular switched-to ADs. Interestingly, besides being often to be switched away, we observed that citalopram and paroxetine were also often switched to during pregnancy (Table 3).

### DDD adjustment of antidepressants before and during pregnancy

For most cases, we observed smaller than 10% change in the mean DDDs of the most continued ADs during pregnancy. Within three groups observed, the DDD adjustment was only significant for sertraline continuers from the low-dose group in which the mean DDDs of this drug increased by 15.4% during pregnancy (*P*=0.025). The highest DD adjustment was for fluoxetine users (+16.7%), yet due to the low sample size the difference in the mean DDDs before and during pregnancy was insignificant (Table 4). On average, the adjustment of mean DDDs before and during pregnancy for the most continued AD seems negligible.

**Table 4:** Comparison between the mean number of DDDs used per day and the DDD adjustment (%) before and during pregnancy for the top five AD continuers, given as overall group and stratified based on high-dose and low-dose groups^$^

		*Overall group*				*High-dose group*				*Low-dose group*			
		*n*	*Mean DDDs (± SD)*	*P value*	*DDD adjustment*^*#*^ *(%)*	*n*	*Mean DDDs (± SD)*	*P value*	*DDD adjustment (%)*	*n*	*Mean DDDs (± SD)*	*P value*	*DDD adjustment (%)*
*Paroxetine*	*Before*	267	1.07 ± 0.47	0.475	−2.8%	177	1.26 ± 0.45	0.252	−4.8%	90	0.71 ± 0.22	0.552	+2.8%
	*During*		1.04 ± 0.50				1.20 ± 0.53				0.73 ± 0.23		
*Citalopram*	*Before*	274	1.09 ± 0.47	0.630	−1.8%	175	1.32 ± 0.43	0.105	−6.1%	99	0.70 ± 0.21	0.083	+10%
	*During*		1.07 ± 0.50				1.24 ± 0.49				0.77 ± 0.34		
*Sertraline*^*$*^	*Before*	136	1.66 ± 0.86	0.566	+3.6%	75	2.27 ± 0.65	0.931	−0.4%	61	0.91 ± 0.31	0.025*	+15.4%
	*During*		1.72 ± 0.86				2.26 ± 0.75				1.05 ± 0.37		
*Venlafaxine*	*Before*	119	0.92 ± 0.48	0.262	−7.6%	39	1.50 ± 0.35	0.125	−10.7%	80	0.64 ± 0.18	0.334	−4.7%
	*During*		0.85 ± 0.48				1.34 ± 0.54				0.61 ± 0.21		
*Fluoxetine*	*Before*	90	1.33 ± 0.62	0.740	−2.3%	67	1.50 ± 0.61	0.504	−4.7%	23	0.78 ± 0.19	0.125	+16.7%
	*During*		1.30 ± 0.59				1.43 ± 0.60				0.91 ± 0.35		

^$^Women belonged to the high-dose group if using ≥ 1 DDD per day (or ≥1.5 DDD for sertraline) before pregnancy and those using < 1 DDD per day before pregnancy belonged to the low-dose group

*Statistically significant if *P* <0.05

^#^Positive value (in %) indicates an increase in the mean DDDs of an AD prescription during pregnancy

## Discussion

In the present study, we observed a rise in the continuation rate of SSRI and SNRI classes during pregnancy from 2001 to 2020. The rate of switching to AD monotherapy during pregnancy was consistently low over time, with SSRIs being the most switched to drugs. All AD drugs investigated did not show substantial changes in the mean DDDs before and during pregnancy, except for sertraline.

On average, we observed that women who received AD medication before pregnancy had a statistically significant difference in their mean age compared to the mean age of pregnancy population in our database. However, the absolute difference in the mean age between two groups is small and does not seem to be of clinical relevance. The continuation rate of AD, mainly the SSRI class, during pregnancy showed an increasing trend over time (see Fig. 2), which aligns with previous reports in the Netherlands showing that the continuation rate of SSRI during pregnancy increased twofold from 19% to 46% (1999–2014) (Molenaar et al. 2020b). SSRIs were relatively new to the market in the early 2000s, in which the effects on pregnancy were not yet fully known. As a result, prescribers may have been reluctant to prescribe an AD. From 2006 onwards, there were increasing experiences with the use of fluoxetine, citalopram, paroxetine, and sertraline during pregnancy as reported in the “Commentaren Medicatiebewaking” reference book (Stichting Health Base 2020). As more experiences have been reported in the last decade (2012 onwards), additional preference is also given to certain SNRIs, such as venlafaxine and duloxetine (NVOG 2021; Bijwerkingen Centrum Lareb 2022).

According to existing recommendations from NVOG and Lareb, a careful assessment plan should be made of the benefits and risks of using an AD during pregnancy for a woman who has already used an AD before conception (NVOG 2012, 2021). When decided to continue the medication, the existing recommendations advise to use the existing medication or opt for another preferred AD. To prevent relapse risk of depression or anxiety disorders, abrupt switching or discontinuing medication during pregnancy is not recommended (NVOG 2012, 2021; Bijwerkingen Centrum Lareb 2021a, 2022).

The SSRI drugs especially sertraline and citalopram were most frequently continued during pregnancy (Table 2). This result is coherent with the existing recommendations (NVOG 2012; Bijwerkingen Centrum Lareb 2021a), and findings from other studies mention citalopram, escitalopram, and sertraline were among the most commonly prescribed AD during pregnancy (Zoega et al. 2015; Bénard-Laribière et al. 2018; Damkier et al. 2018; Donald et al. 2021). In particular, paroxetine is frequently prescribed, although a slightly increased risk of fetal heart defects cannot be ruled out (NVOG 2012).

Bupropion was the most often discontinued AD during pregnancy over the study period, at 84%. This result suggests that bupropion may have been prescribed as an aid in smoking cessation, which is one of the labeled indications of use for this drug (Zorginstituut Nederland 2022d). This therapeutic goal can also be achieved without pharmacotherapy. Bupropion can be discontinued, or when necessary, it can be switched to another safer drug, such as an SSRI (Bijwerkingen Centrum Lareb 2021b).

The switching rate of ADs during pregnancy was low and steady at around 1.8% over the study period. This low rate was expected because most guidelines advise patients to continue the same treatment in instances in the event a patient is psychiatrically stable and responsive, the treatment is effective, or the patient has a history of relapse/recurrence off medication (Kim et al. 2010; NVOG 2012, 2021; Molenaar et al. 2018; Bijwerkingen Centrum Lareb 2021a, 2022). Furthermore, our findings are similar to results in Nordic population (Denmark, Iceland, Norway, Sweden) mentioning a low proportion of switchers (1.8%) in early pregnancy (Zoega et al. 2015).

All switchers changed to another AD monotherapy during pregnancy, of which 82% of the cases switched from an SSRI to another. Additionally, most women who previously used an SSRI in combination with other ADs switched to another SSRI during pregnancy. More in-depth, fluoxetine, sertraline, and citalopram became the most preferred drugs to switch to. This choice seems logical as these SSRI drugs are slightly preferred during pregnancy over the SNRI or TCA drugs in the national guidelines (NVOG 2012; Bijwerkingen Centrum Lareb 2021a). This finding is also similar to previous studies which reported sertraline and citalopram as the preferred drugs to switch to (Bénard-Laribière et al. 2018; Molenaar et al. 2020b).

The present study also observed that 82% of switching were between different ADs in the same class of SSRI. For several switchers, paroxetine or citalopram was switched from, while for others, these drugs were switched to (Table 3). This trend could indicate that the switching observed during pregnancy is not necessarily a result of a perceived risk of drug use to the mother and unborn child during pregnancy, but perhaps it is caused by a woman developing intolerance or poor response to the initial medication, unacceptable side effects, or an intention to breastfeed after delivery. When switching is necessary, the initial medication can be tapered-off followed by a drug-free washout interval, directly switched with the second AD or cross-tapered between them simultaneously (Keks et al. 2016; Folsche et al. 2021).

The absolute difference in the mean DDDs for all ADs studied was less than 10% change before and during pregnancy for all ADs studied, indicating that the observed DDD adjustment was meager, mostly comparable, and considered to be clinically irrelevant. A similar result of slight difference (<10%) in the mean DDDs before and during pregnancy among AD continuers has been reported in Norway (Trinh et al. 2022). The only exception AD with a significant adjustment of DDD is sertraline. Those with a low DDD before pregnancy had a dose increase by around 15% during pregnancy. During pregnancy, it was reported that the metabolism and clearance for sertraline may be increased and lower AUC and Cmax were observed particularly between the 2^nd^ and 3^rd^ trimesters which results in the potential need for dose increases during pregnancy (Sit et al. 2008; Freeman et al. 2008; Schoretsanitis et al. 2020; George et al. 2020). However, maternal concentrations vary greatly during pregnancy partly explained by interindividual variability in hepatic drug metabolizing cytochrome P450-enzyme activity (Heinonen et al. 2021). As such, even though the reference range of sertraline is broad (10–150 ng/mL), it is less likely that the increased dose may affect efficacy and pregnancy outcomes (Hiemke et al. 2018; Schoretsanitis et al. 2020). It is also important to note that despite having a wide range of recommended doses (50 to 200 mg orally once daily), the DDD of sertraline prescribed to a patient may be impacted by the diagnosis and symptom domain (WHOCC 2022b; Zorginstituut Nederland 2022c).

A different pattern of dose adjustment was observed among women taking a high or low dose of ADs before conception. The group of women taking a high dose had a decreased mean DDDs during pregnancy. In contrast, those taking a low dose had an increased daily dose (except for venlafaxine). Despite these patterns being clearly observed, a regression to the mean phenomenon might occur due to natural variation of mean DDDs, causing the observed changes within the high and low-dose groups to move toward the mean value of DDDs (Bland and Altman 1994).

### Limitations and strengths

It should be noted that our findings should be interpreted with caution. First, as the IADB.nl database solely records dispensing information, the actual utilization is not documented. Hence, women who discontinue their medication while still receiving prescriptions may be misclassified as AD continued users. Second, the pregnancy database does not store information regarding the underlying diagnosis for which an AD medication is prescribed. Therefore, the indication of AD use in pregnancies studied cannot be considered in our analysis. Finally, as the conception date is uncertain, we used the theoretical conception date to be set at 39 weeks before the child’s birthdate. A small proportion of women may be misclassified as a continuer, a discontinuer, or a switcher due to preterm or postterm birth. Nevertheless, the present study investigated a topic that has not been studied widely in the Netherlands by detailing the trends of AD use in the past twenty years, particularly the switching patterns and dose adjustments around conception.

As the population in the IADB database represents the Dutch population (Visser et al. 2013; Sediq et al. 2018), the findings of this study are generalizable to the entire country. This study also spans a longer study period to analyze AD prescriptions’ switching patterns and dose adjustments over time. Lastly, the observed trends in current study were comparable to findings from other studies in France and four Nordic countries (Zoega et al. 2015; Bénard-Laribière et al. 2018; Trinh et al. 2022), indicating external validity of the study outcomes.

## Conclusion

A twofold increase in the continuation rate of SSRIs during pregnancy was observed between 2001 and 2020 showing that those prescribed before conception are considered safe for the mother and unborn child. The proportion of switching between ADs was consistently low over time and the daily doses of ADs received before and during pregnancy were comparable, which may indicate that women included in this study were well-adjusted to their prepregnancy medication.

Most of the findings in this study are in accordance with the national guidelines that recommend two principal elements. First, the use of ADs could be continued during pregnancy if women are already benefited and stable with their medication. Second, switching between antidepressants during pregnancy without solid reasons/rationale should be avoided; if necessary, switching should be initiated before pregnancy.

## Supplementary information

ESM 1 (DOCX 15 kb)

## Data Availability

The dataset for this manuscript is not publicly available due to the IADB data protection policy. Request to access the database should be directed to the corresponding author upon reasonable request.
