# Supplementary material for: Switching pattern and dose adjustment of antidepressants before and during pregnancy
Source: Arch Womens Ment Health. 2023 Aug 5;26(5):685–96. doi: 10.1007/s00737-023-01355-8 (PMC10491541; doi:10.1007/s00737-023-01355-8)
Supplement: Supplementary file 1 — (DOCX 15 kb) [file 737_2023_1355_MOESM1_ESM.docx]

**SUPPLEMENTARY INFORMATION**

**S1 Table** Antidepressant drugs excluded from the cohort selection

| *Antidepressants with low number of users (≤2 pregnancies exposed)* | | |
| --- | --- | --- |
| mianserin | oxitriptan | doxepin |
| phenelzine | hyperici Herba | tranylcypromine |
| maprotiline | dosulepine | moclobemide |
| trimipramine | nefazodone |  |

**S2 Table** Comparison of continuation, discontinuation, and switching rates of antidepressant users before and during pregnancy from 2001 to 2020

|  | *Continuation rate*  *n (%)* | *Discontinuation rate*  *n (%)* | *Switching rate*  *n (%)* |
| --- | --- | --- | --- |
| *Total period* | 1,172 (44.1) | 1,435 (54.0) | 48 (1.8) |
| *2001-2005* | 113 (25.1) | 329 (72.9) | 9 (2.0) |
| *2006-2010* | 209 (35.8) | 362 (62.0) | 13 (2.2) |
| *2011-2015* | 413 (47.7) | 443 (51.2) | 9 (1.0) |
| *2016-2020* | 437 (57.9) | 301 (39.9) | 17 (2.3) |
